# Mitochondrial Hormesis links nutrient restriction to improved metabolism in fat cell

**DOI:** 10.18632/aging.100832

**Published:** 2015-10-28

**Authors:** Daniele Lettieri Barbato, Giuseppe Tatulli, Katia Aquilano, Maria R. Ciriolo

**Affiliations:** ^1^ Department of Biology, University of Rome “Tor Vergata”, 00133 Rome, Italy; ^2^ Università Telematica San Raffaele Roma, 00166, Rome, Italy; ^3^ IRCCS San Raffaele Roma, 00163, Rome, Italy

**Keywords:** mitochondria, adipose tissue, aging, FoxO1, energy metabolism, longevity

## Abstract

Fasting promotes longevity by reprogramming metabolic and stress resistance pathways. However, although the impact on adipose tissue physiology through hormonal inputs is well established, the direct role of fasting on adipose cells is poorly understood. Herein we show that white and beige adipocytes, as well as mouse epididymal and subcutaneous adipose depots, respond to nutrient scarcity by acquiring a brown-like phenotype. Indeed, they improve oxidative metabolism through modulating the expression of mitochondrial-and nuclear-encoded oxidative phosphorylation genes as well as mitochondrial stress defensive proteins (UCP1, SOD2). Such adaptation is placed in a canonical mitohormetic response that proceeds via mitochondrial reactive oxygen species (^mt^ROS) production and redistribution of FoxO1 transcription factor into nucleus. Nuclear FoxO1 (^n^FoxO1) mediates retrograde communication by inducing the expression of mitochondrial oxidative and stress defensive genes. Collectively, our findings describe an unusual white/beige fat cell response to nutrient availability highlighting another health-promoting mechanism of fasting.

## INTRODUCTION

In the past century, the incidence of chronic age-related diseases, particularly obesity and type 2 diabetes, has increased dramatically [1] and it has been suggested that adipose tissue might have a central role in these pathologies [2]. Visceral white adipose tissue (WAT) progressively expands during life thus participating in the metabolic perturbation occurring during aging [3-5]. Accordingly, interventions that limit WAT enlargement are associated with enhanced health and life span [6]. Dietary regimens that are characterized by a reduced nutrient or calorie intake lead to visceral fat lowering and delay aging [6]. Among these dietary approaches, fasting can be easily managed by ingesting no or minimal amounts of nutrients and calories for brief periods [7]. Fasting promotes powerful changes in metabolic processes, suggesting its clinical application in several metabolic disorders. In humans, fasting typically results in a 20% or greater decrease in serum glucose, insulin and insulin-like growth factors setting a metabolic mode in which free fatty acids are used as energy sources [7]. Controlled fasting retards aging and some age-related diseases (e.g type 2 diabetes) by shared mechanisms involving improved cellular stress adaptations [7]. Although the *gerosuppressant* signaling pathways induced by fasting are well characterized in several species and tissues [6-7], evidence on WAT is weakly explored yet.

WAT is commonly considered as an energetic rheostat fasting can be easily managed by ingesting no or in the body releasing fatty acids through lipolysis, minimal amounts of nutrients and calories for brief whereas brown adipose tissue (BAT) is the site of periods [7]. Fasting promotes powerful changes in meta-mitochondrial energy dissipation through thermogenesis. Recent observations demonstrate that white and beige adipocytes may develop a “brown-like” phenotype leading to favourable effects on overall metabolism and possibly reducing the risk of age-related pathologies [8]. Canonically, lipolysis as well as thermogenesis ignition is controlled by hormonal input mediated by cAMPK/PKA signaling cascade. However, it is emerging that fat cells can directly sense environmental changes in a cell-autonomous manner [9]. This suggests that a lineage of fat cells may develop a peculiar adaptive capacity involving alternative signaling pathways to the canonical hormonal stimuli.

Several studies demonstrated that in various model organisms the life extending effects of nutrient restriction are consequence of improved oxidative metabolism [10]. In conflict with the Harman's free radical theory of aging these effects may be mediated by transient mitochondrial ROS (^mt^ROS) production promoting a retrograde signaling that triggers the induction of nuclear-encoded mitochondrial stress defensive proteins and ultimately leads to a long-term improved redox state [11-12]. This type of retrograde response has been named *mitohormesis*, and may be relevant to the health-promoting effects of nutrient shortage in model organisms [10]. Coherently, abrogation of ^mt^ROS by antioxidants impairs the life-extending program and health-promoting capabilities of nutrient restriction thus highlighting ^mt^ROS as essential signaling molecules [13].

The nutrient sensing FoxO1 is a crucial metabolic regulator in several tissues and commonly placed into longevity pathways [4, 14-15]. Notably, treatment with a compound mimicking nutrient restriction (nicotinamide riboside) elicits FoxO1 nuclear accumulation and this represents a key event promoting longevity by transcription of mitochondrial defensive genes [16]. In fat cells, mild redox imbalance promotes nuclear FoxO1 (nFoxO1) redistribution inducing adipose triglyceride lipase (ATGL), lysosomal acid lipase (Lipa) and enhanced mitochondrial oxidative capacity [4, 17].

In this report, we propose that the health benefits of fasting may also rely on its capability to favour brown-like changes in WAT via the improvement of cellular metabolism and antioxidant equipment. Further, we demonstrate that WAT adipocytes exploit nutrient-sensitive mtROS/nFoxO1 retrograde signaling as alternative pathway to boost their mitochondrial functionality independently of adrenergic cascade.

## RESULTS

### Nutrient restriction leads to mitonuclear OxPHOS imbalance concomitantly to mitochondrial remodeling in white and beige adipose cells

White (WAT) and brown (BAT) adipose tissue display a diverse mitochondrial OxPHOS equipment, which specifically reflects the cellular and tissue functionality.

**Figure 1:**
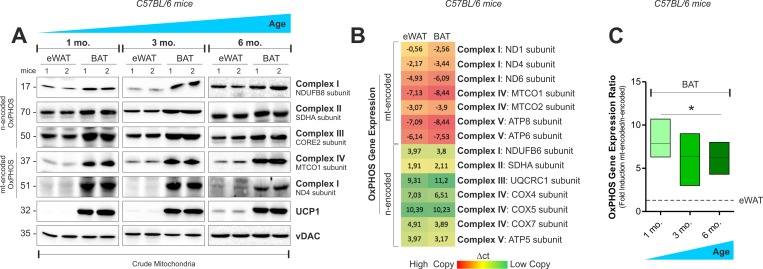
OxPHOS gene expression in mouse white and brown fat (**A**) mtDNA and nDNA-encoded OxPHOS proteins analyzed by Western blot (n=2 mice per group). (**B**) Heat map of mtDNA-and nDNA-encoded OxPHOS genes assayed by RT-qPCR in 4 months old mice (mean value of n=4 mice per group). (**C**) OxPHOS gene expression ratio evaluated by calculating the ratio between mtDNA-and nDNA-encoded OxPHOS genes considered in (**B**) (n=4 mice per group). vDAC served as loading control. Bar graphs are expressed as mean ±S.D. (*p<0.05 vs eWAT).

**Figure 2:**
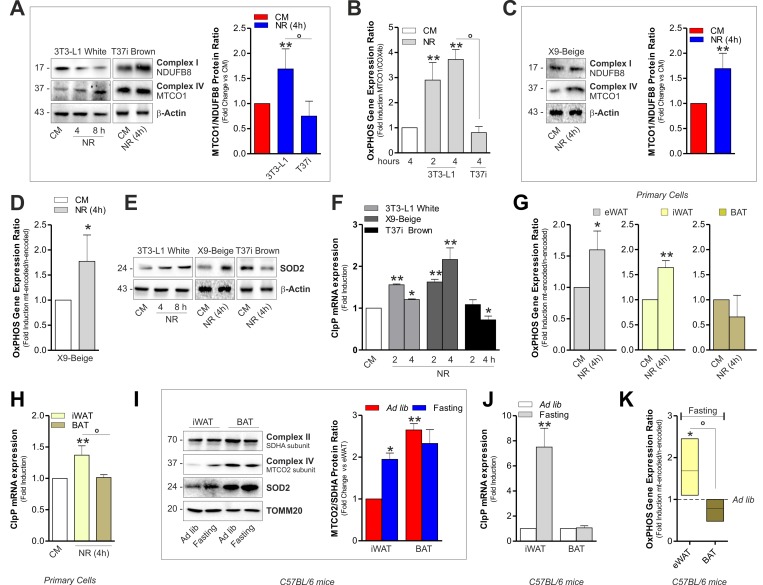
Mitonuclear OxPHOS imbalance and mitochondrial stress response is elicited in white and beige adipocytes upon starvation (**A**) OxPHOS protein ratio (*right panel*) evaluated by calculating the ratio between ^mt^DNA-and ^n^DNA-encoded OxPHOS proteins after Western blot (*left panel*) followed by densitometric analysis. (**B**) OxPHOS gene expression ratio evaluated by RT-qPCR. (**C**) OxPHOS protein ratio evaluated as described in (A). (**D**) OxPHOS gene expression ratio evaluated by calculating the ratio between ^mt^DNA-(MTCO1 and ATP6) and ^n^DNA-encoded (SDHA and Cox4b) OxPHOS mRNAs. (**E, F**) Mitochondrial stress response assessed by Western blot analysis of SOD2 (E) and through RT-qPCR of ClpP (F). (**G**) OxPHOS gene expression ratio evaluated as described in (D). (**H**) mRNA levels of ClpP measured by RT-qPCR. (**I, J**) OxPHOS protein ratio (I, *left panel*) evaluated as described in (A). SOD2 protein (I) and mRNA levels of ClpP (J) evaluated by Western blot and RT-qPCR, respectively (n=4 mice per group). (**K**) OxPHOS gene expression ratio evaluated as described in (D) (n=4 mice per group). Actin and TOMM20 served as loading controls. Bar graphs are expressed as mean ±S.D. (n=4; *p<0.05; **p<0.01 vs CM or *ad libitum* fed mice; °p<0.05). NR: nutrient restriction; CM: complete medium.

Accordingly, in BAT crude mitochondria from 1, 3 and 6 months old mice, we observed higher OxPHOS protein levels than epididymal WAT (eWAT) (Fig. 1A and Fig. S1A). In order to compare the transcriptional level of mitochondrial ^mt^DNA- and nuclear ^n^DNA-encoded OxPHOS genes in white and brown adipose depots, we performed an OxPHOS gene expression array. As showed in Fig. 1B and S1B, both eWAT and BAT revealed higher expression of ^mt^DNA- than ^mt^DNA-encoded OxPHOS mRNA. However, the transcriptional efficiency of BAT (Fig. 1C and Fig. S1C) and T37i brown adipocytes (Fig. S1D) was mainly shifted toward ^mt^DNA-encoded OxPHOS genes. Given that mitonuclear imbalance is involved in the adaptive response to metabolic requirements [18-19], we analyzed whether it could occur in adipocytes upon nutrient restriction (NR). As showed in Fig. 2A and Fig. S1E, NR leads to mitonucle ar protein imbalance in 3T3-L1 adipocytes, as evidenced by the increased ratio between the ^mt^DNA-and the ^n^DNA-encoded OxPHOS proteins. We also revealed a significant increase in the mRNA ratios between ^mt^DNA-encoded MTCO1 and ^n^DNA-encoded COX4b (Fig. 2B). The analyses of other OxPHOS gene expression ratios revealed the same trend (Fig. S1F). The results obtained by the calculation of the OxPHOS gene expression ratio revealed increased mitochondrial transcription efficiency in white adipose depots (Fig. S1G). Conversely, T37i adipocytes had not increased ratios between MTCO1 and ^n^DNA-encoded NDUFB8 proteins (Fig. 2A) as well as MTCO1 and Cox4b mRNAs (Fig. 2B). Furthermore, also beige adipose cells up-regulated the ratio between ^mt^DNA-and ^n^DNA-encoded proteins and mRNAs after NR (Fig. 2C and 2D). The disturbance of the delicate mitonuclear balance triggers the induction of ^n^DNA-encoded mitochondrial stress factors such as antioxidant enzyme SOD2 and protease ClpP [16, 20]. As expected, we found that NR increased SOD2 and ClpP levels in 3T3-L1 and X9 but not in T37i adipocytes, where on the contrary SOD2 and ClpP levels were diminished (Fig. 2E and 2F). Similarly to immortalized adipocytes, primary adipocytes displayed an increased ratio between ^mt^DNA-and ^n^DNA-encoded genes (Fig 2G) as well as increased ClpP mRNA levels (Fig 2H). Primary adipocytes derived from BAT did not show this trend (Fig 2G and 2H). Finally, we investigated the mitochondrial adaptive response in adipose tissues of 20 h fasted mice. An increased ratio between ^mt^DNA-encoded MTCO2 and ^n^DNA-encoded SDHA proteins as well as an accumulation of SOD2 (Fig 2I) and ClpP (Fig 2J) was disclosed in iWAT but not in BAT crude mitochondrial fractions. Expectedly, 20 h fasting led to an increased mRNA ratio between ^mt^DNA-and ^n^DNA-encoded OxPHOS genes in eWAT (Fig 2K) with respect to *ad libitum* fed mice.

**Figure 3:**
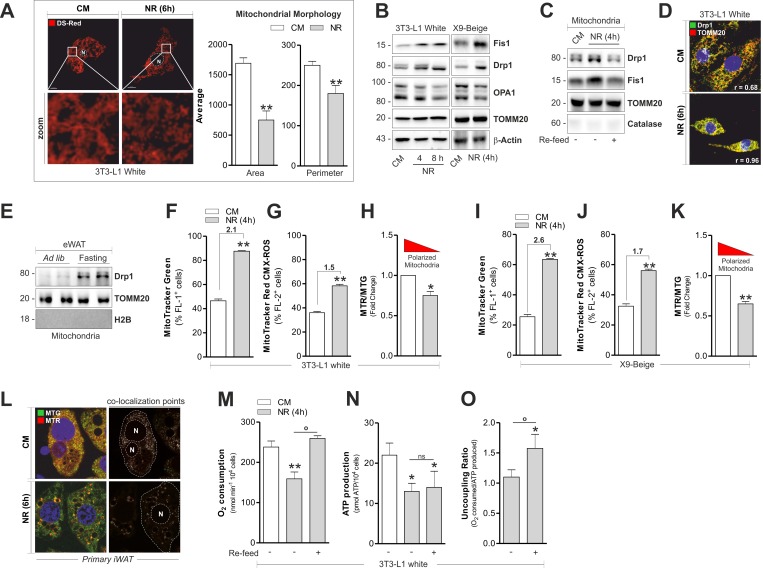
Fragmentation and altered functionality occurs in mitochondria of white and beige adipose cells after starvation (**A-C**) Mitochondrial fragmentation assessed by analyzing mitochondrial morphology through confocal microscopy in cells transfected with mitochondrial Ds-Red fluorescent protein (A) or by analyzing the content of Drp1, Fis1 and OPA1 through Western blot (B, C). (**D**) Fragmented mitochondria detected through confocal microscopy after co-staining with Drp1 and TOMM20 antibodies. (**E**) Mitochondrial fragmentation assessed as described in (B) in crude mitochondria (n=4 mice per group). (**F-K**) Mitochondrial amount and membrane potential quantified by MitoTracker Green (F, I) and MitoTracker Red CMX-ROS (G, J) by cytofluorimetric analysis, respectively. Polarized mitochondria determined by calculating Red-to-Green ratio (MTR/MTG) (H, K). (**L**) Polarized mitochondria detected through confocal microscopy after staining with MitoTracker Green and MitoTracker Red CMX-ROS. Co-localization points indicate polarized mitochondria. (**M-O**) Polarographic recording of oxygen consumption (M) and cheminoluminescent assay of ATP content (N) under NR and 1h after nutrient refill (Re-feed) with complete culture medium. Mitochondrial uncoupling determined by calculating the ratio between O_2_ consumption and ATP production (O). Bar graphs are expressed as mean ±S.D. (n=3; *p<0.05; **p<0.01 vs CM; °p<0.05). Actin, TOMM20, H2B and catalase staining served as loading controls or for assessing the purity of cell protein fractions. NR: nutrient restriction; CM: complete medium.

Mitochondria undergo continuous cycles of selective fusion and fission, and nutrients as well as hormones efficiently affect these processes [21]. Although NR increases oxidative metabolism in white adipocytes [4], its effect on adipocyte mitochondrial dynamic is still unknown. Through morphometric analysis of confocal microscopy images we observed that mitochondria of 3T3-L1 adipocytes appeared higher in number but smaller in per imeter and area, implying the occurrence of mitochondrial fragmentation (Fig 3A). In line with this hypothesis, 3T3-L1 adipocytes accumulated Drp1 and Fis1 in total protein extracts (Fig 3B) as well as in crude mitochondrial fractions (Fig 3C), while the canonical marker of mitochondrial fusion OPA1 underwent a progressive reduction (Fig 3B). The mitochondrial dynamic observed in 3T3-L1 cells was not accompanied by variations in total levels of the mitochondrial protein translocase TOMM20 (Fig 3B). Nutrient refill with complete medium (CM) was able to restore the basal level of Drp1 and Fis1 localized at mitochondrial level in 3T3-L1 adipocytes (Fig 3C). Differently, no significant changes in mitochondrial fragmentation markers were detected in T37i adipocytes subjected to NR (Fig S1H). By confocal microscopy analysis we revealed a higher co-localization degree (Pearson coefficient, r = 0.96) between Drp1 and TOMM20 in 3T3-L1 adipocytes upon NR (Fig 3D) and increased mitochondrial fission was also observed in starved X9 adipocytes (Fig 3B). These results were recapitulated in crude mitochondria isolated from 20 h fasted mice in which we detected an increased level of Drp1 in eWAT mitochondria (Fig 3E). Next we quantified mitochondrial amount and membrane potential (ΔΨM) by staining 3T3-L1 and X9 adipocytes with MitoTracker Green (MTG) and MitoTracker Red (MTR), respectively. Interestingly, NR increased the degree of MTG positive 3T3-L1 (Fig 3F) and X9 adipocytes (Fig 3I). An increase of ΔΨM was evidenced, although at lesser extent with respect to mitochondrial number (Fig 3G and 3J). Such ΔΨM increase was apparent, as established by normalizing MTR fluorescence (sensitive both ΔΨM and mitochondrial number) with MTG value, which is sensitive only to mitochondrial number. In particular, as reported in Fig 3H and 3K, the calculation of Red-to-Green ratio (MTR/MTG) evidenced that starved adipocytes undergo mitochondrial depolarization. The same results were obtained by staining mitochondria with the cardiolipin fluorescent probe nonyl acridine orange (indicative of mitochondrial number) and tetramethyl rhodamine (sensitive to ΔΨM) (Fig S2A). In line with these data, primary beige adipocytes displayed a reduction in co-localization points between MTG and MTR after NR (Fig 3L). Mitochondrial changes were also accompanied by a diminished O_2_ consumption along with a drop of ATP level in 3T3-L1 white adipocytes (Fig 3M and 3N). To verify whether such variations were due to the deprivation of nutrients, we refill cells with CM. Under this condition, we found restoration of O_2_ consumption without a concomitant recovery of ATP (Fig 3M and 3N), suggesting the occurrence of an uncoupling process (Fig 3O), accompanied by improved OxPHOS machinery (Fig 2).

### Starvation induces UCP1 independently of hormonal signaling cascade

**Figure 4:**
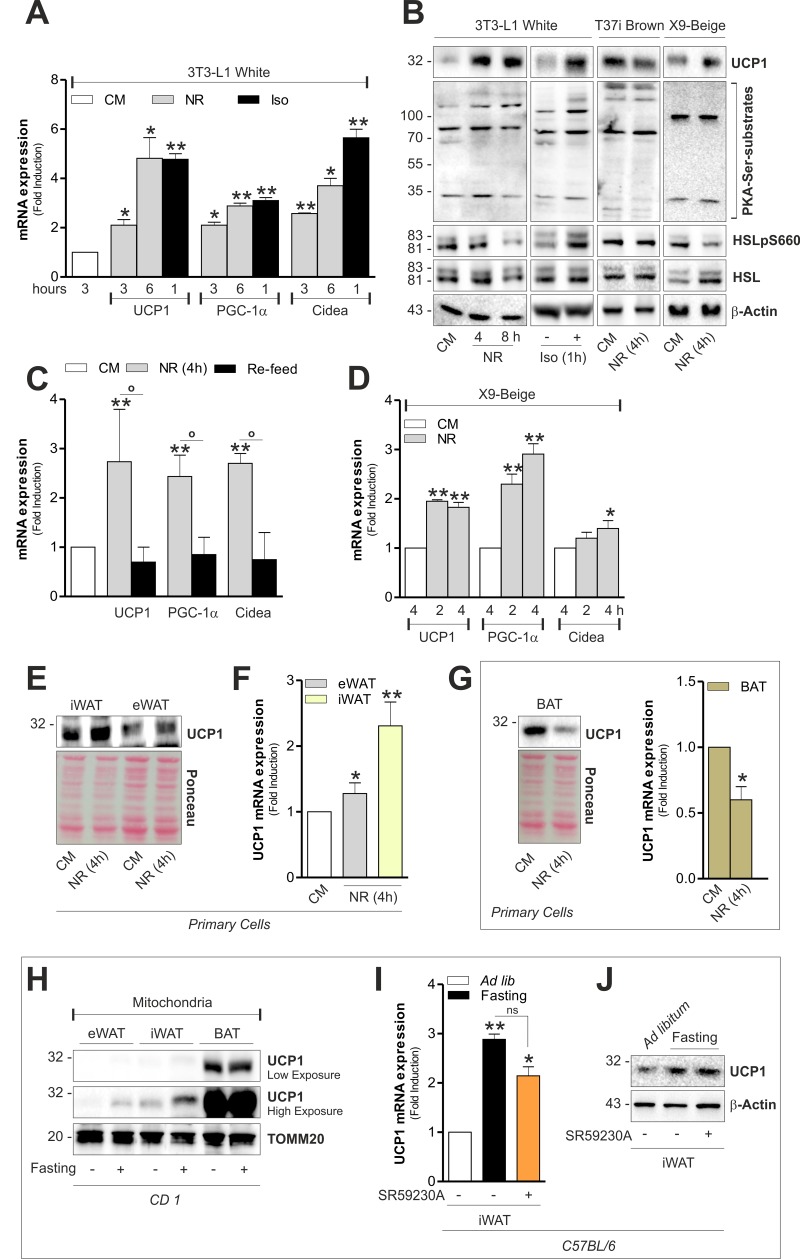
White and beige adipose cells undergo brown fat-like changes independently of hormones (**A**) Induction of brown-related genes evaluated by analyzing the mRNA expression of UCP1, PGC-1α and Cidea through RT-qPCR. (**B**) Protein level of UCP1, HSL, phospho-active HSL (HSLpS660) and PKA serine substrates assayed through Western blot analysis in total cell lysates. (**C**) Induction of brown-related genes evaluated as described in (**A**) after 1h nutrient refill with complete culture medium (Re-feed). (**D**) Induction of brown-related genes evaluated as described in (**A**). (**E-G**) UCP1 protein and mRNA levels measured by Western blot (**E**, **G**) and RT-qPCR (**F**, **G**) analysis. (**H**) UCP1 protein level analyzed by Western blot in crude mitochondria (pool of n=6 mice per group). (**I, J**) UCP1 mRNA (**I**) and protein (J) levels measured by RT-qPCR and Western blot analysis (n=6 mice per group). Actin, TOMM20 and Ponceau Red staining served as loading controls. Bar graphs are expressed as mean ±S.D. (n=4; *p<0.05; **p<0.01 vs CM or *ad libitum* fed mice; °p<0.05). NR: nutrient restriction; CM: complete medium; Iso: isoproterenol.

Augmented mitochondrial fragmentation and uncoupling have been identified as key features that accompany the browning process [22]. As reported in Fig 4A, the expression of brown fat-related markers were increased in 3T3-L1 adipocytes upon NR to levels comparable to those reached upon treatment with the β-adrenoreceptor agonist isoproterenol. Concomitantly, UCP1 protein levels increased both during NR and isoproterenol treatment (Fig 4B). Furthermore, the newly synthetized UCP1 resulted localized into mitochondria (Fig S2B). To more specifically link the development of a brown-like phenotype to NR, we cultured starved 3T3-L1 adipocytes with CM (re-feed). As demonstrated in Fig 4C, nutrient refill was effective in reverting the up-regulation of PGC-1α, UCP1 and Cidea. In accordance with the above-described data, X9 adipocytes quickly responded to NR by up-regulating (Fig 4E and 4F). Conversely, we revealed a down-brown-related markers (Fig 4B and 4D). UCP1 was regulation of UCP1 level both in immortalized T37i also induced in primary beige and white adipocytes (Fig 4B) and primary brown adipocytes (Fig 4G).

Generally β-adrenoreceptor stimulation culminates in UCP1 expression and lipolysis in adipose cells by the activation of cAMP/PKA pathway [22]. To study the involvement of cAMP/PKA signaling cascade during NR, we checked the phosphorylation state of hormone sensitive lipase (HSL) on PKA-target serine (i.e. ser^660^). Differently from isoproterenol stimulation, NR did not induce changes in phospho-active levels of HSL on ser^660^ (HSLpS660) (Fig 4B), suggesting that 3T3-L1 and X9 adipocytes develop a brown-like phenotype independently of adrenergic stimulation. We next analyzed UCP1 levels in mitochondria isolated from tissues of *ad libitum* fed or fasted mice. As showed in Fig 4H, 20 h fasting was efficient in promoting UCP1 up-regulation only in white and beige adipose depots. However, given that in an *in vivo* system, the occurrence of noradrenaline modulation could affect the UCP1 level in adipose tissue, we intraperitoneally injected a selective β3 adrenoreceptor antagonist (SR59230A) prior to fasting. Notably, although SR59230A was effective in limiting fat mass loss during fasting (Fig S2C), it did not completely restrain UCP1 increase in iWAT (Fig 4I and 4J).

### ^mt^ROS trigger mitochondrial responses in white and beige adipocytes

**Figure 5:**
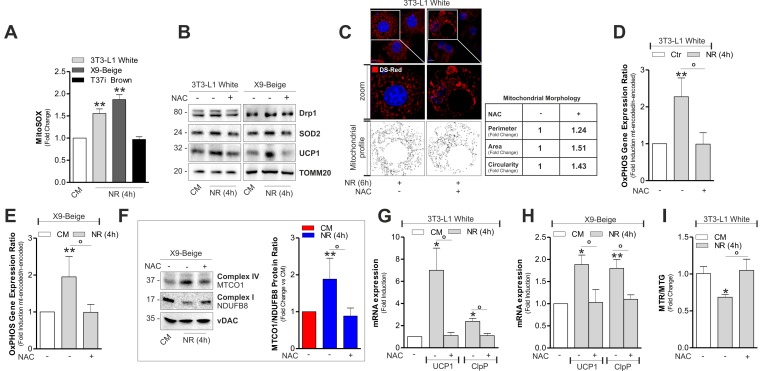
^mt^ROS are involved in the induction of mitonuclear stress response and uncoupling (**A**) Cytofluorimetric detection of ^mt^ROS after staining with MitoSOX. (**B**) Protein levels of Drp1, SOD2 and UCP1 analyzed by Western blot. (**C**) Mitochondrial fragmentation detected through confocal microscopy in cells transfected with mitochondrial Ds-Red fluorescent protein and treated or not with NAC. (**D, E**) OxPHOS gene expression ratio evaluated by calculating the ratio between ^mt^DNA-encoded (MTCO1 and ATP6) and ^n^DNA-encoded (SDHA and Cox4b) mitochondrial mRNAs after RT-qPCR in cells treated or not with NAC. (**F**) OxPHOS protein ratio (*right panel*) evaluated by calculating the ratio between ^mt^DNA-encoded (MTCO1) and ^n^DNA-encoded (NDUFB8) mitochondrial proteins (*left panel*) after Western blot followed by densitometric analysis. (**G, H**) mRNA levels of UCP1 and ClpP measured through RT-qPCR in cells treated or not with NAC. (**I**) Cytofluorimetric detection of mitochondrial amount and membrane potential through MitoTracker Green (MTG) and MitoTracker Red CMX-ROS (MTR) staining, respectively. Polarized mitochondria determined by calculating Red-to-Green ratio (MTR/MTG). TOMM20 and vDAC staining served as loading control. Bar graphs are expressed as mean ±S.D. (n=4; *p<0.05; **p<0.01 vs CM; °p<0.05). NR: nutrient restriction; CM: complete medium.

Mitochondrial ROS (^mt^ROS) production appears to be a tightly regulated process that is essential for tuning the magnitude or effectiveness of the mitochondrial adaptive response [23]. NR is able to impinge ^mt^ROS that are key mediators of the signal transduction pathway culminating in the metabolic adaptation of 3T3-L1 adipocytes [4]. Similarly to 3T3-L1, we found an increased ^mt^ROS production also in X9 cells, while T37i adipocytes were unresponsive (Fig 5A). Interestingly, the lack of oxidative phenomena in brown adipocytes observed after ^mt^ROS-generating treatments (i.e. fasting) could be linked to basal higher mitochondria antioxidant defensive proteins (SOD2 and UCP1) in BAT with respect to WAT (Fig S3B). Successively, to dissect the role of ^mt^ROS in orchestrating mitochondrial stress response, we treated 3T3-L1 and X9 adipocytes with the antioxidant *N*-acetyl cysteine (NAC) prior to NR. As expected, NAC was able to prevent ^mt^ROS production both in 3T3-L1 and X9 adipocytes (Fig S3C) and it was effective in limiting SOD2 and UCP1 proteins up-regulation (Fig 5B). These results were recapitulated by overexpressing SOD2 [SOD2(+)] in 3T3-L1 adipocytes (Fig S3D). Furthermore, antioxidant-boosting strategies (NAC treatment or SOD2 overexpression) were also effective in limiting Drp1 induction (Fig 5B and S3D) and mitochondrial fragmentation (Fig 5C). Our data describe the ability of ^mt^ROS also in governing mitochondrial adaptive responses to NR in white and beige adipocytes. Indeed, NAC treatment prevented the alteration of the ratio between ^mt^DNA-and ^n^DNA-encoded imbalance both in 3T3-L1 (Fig 5D) and X9 (Fig 5E and 5F) adipose cells. NAC was also able to prevent UCP1 and ClpP mRNA elevation in 3T3-L1 (Fig 5G) and X9 adipocytes (Fig 5H). Mitochondrial depolarization in 3T3-L1 adipocytes was also dampened (Fig 5I) in line with unchanged UCP1 levels.

### ^n^FoxO1 mediates the retrograde communication enforcing mitochondrial adaptive responses in white and beige adipocytes

**Figure 6:**
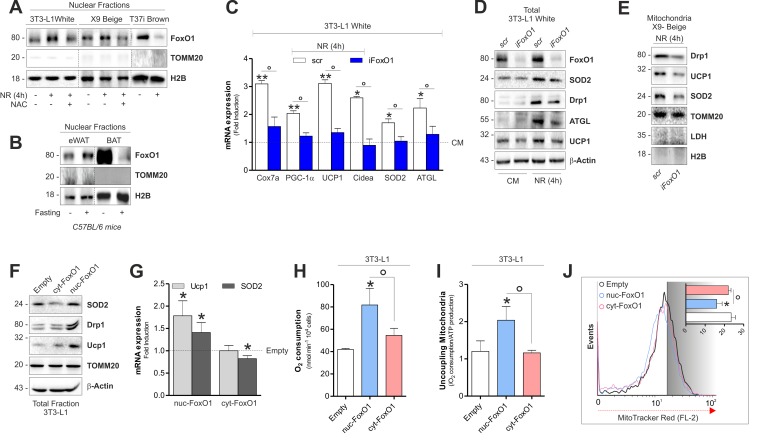
FoxO1 modulates mitochondrial stress response and expression of brown-related genes in white and beige adipose cells (**A**) Protein levels of FoxO1 analyzed by Western blot in nuclear fractions of starved adipocytes treated or not with NAC. (**B**) Protein levels of FoxO1 analyzed by Western blot in nuclear fractions (n=4 mice per group). (**C-E**) mRNA (C) and protein (D, E) levels of brown fat-related genes analyzed by RT-qPCR or Western blot in starved cells transfected with a scramble or FoxO1 RNAi. (**F, G**) Protein (F) and mRNA (G) expression levels of brown fat-related genes analyzed by Western blot or RT-qPCR in cells transfected with plasmids containing nuc-FoxO1 or cyt-FoxO1 cDNAs. (**H, I**) Polarographic recording of oxygen consumption (H) and cheminoluminescence assay of ATP content (I) in cells transfected with plasmids containing nuc-FoxO1 or cyt-FoxO1 cDNAs. Mitochondrial oxidative phosphorylation efficiency determined by calculating the ratio between ATP production and O_2_ consumption (P:O ratio). (**J**) Cytofluorimetric detection of mitochondrial membrane potential after staining cells with MitoTracker Red CMX-ROS. Actin, H2B, TOMM20 and LDH staining served as loading control. Bar graphs are expressed as mean ±S.D. (n=4; *p<0.05 vs CM or Empty; **p<0.01 vs CM; °p<0.05). NR: nutrient restriction; CM: complete medium.

We previously showed that ^mt^ROS produced during NR in 3T3-L1 adipocytes are key mediators in forcing FoxO1 into nuclear compartment [4]. In line with this assumption, NR elicited the migration of FoxO1 into nuclei both in white and beige fat cells and NAC sup-plementation was able to restrain FoxO1 redistribution (Fig 6A and 6B). In the opposite manner, brown fat cells displayed a diminished ^n^FoxO1 level upon NR (Fig 6A and 6B). Nuclear FoxO1 is the up-stream mediator of transcription of metabolic genes conferring resistance to NR [17, 24]. Furthermore, nuclear form of FoxO1 induces PGC-1α, UCP1, SOD2 [25] as well as ATGL gene transcription [4]. To better understand whether FoxO1 was implicated in the retrograde communication of starved adipocytes, we initially down-regulated it in 3T3-L1 adipocytes (iFoxO1). As described in Fig 6C and 6D, an impaired up-regulation of brown fat-related genes in white iFoxO1 adipocytes, was observed. Concomitantly, we found that upon NR iFoxO1 adipocytes had lower level of mitochondrial fragmentation marker Drp1 than controls (Fig 6D). Similarly, down-regulation of FoxO1 in X9 beige adipocytes resulted in decreased level of Drp1, SOD2 and UCP1 (Fig 6E).

In white adipocytes, mitochondrial complex I inhibition by non-toxic amount of rotenone induces ^mt^ROS production prompting a pseudo-starvation response [4]. In line with our previous findings, rotenone impinges FoxO1 into nuclei of white adipocytes (Fig S4A) and this event is associated with SOD2 and UCP1 induction (Fig S4B and S4C). Differently, in T37i brown fat cells we did not observe the same responses (Fig S4A and S4B).

To finally implicate nuclear redistribution of FoxO1 in the uncoupling adaptive response of white adipocytes, we transfected 3T3-L1 adipocytes with a form of FoxO1 that is retained within the cytoplasm (cyt-FoxO1, GFP-tagged) or with a constitutively active form of FoxO1 (Myc-tagged) that is specifically localized within nuclei (nuc-FoxO1) (Fig S4D). Western blot analysis indicated that 3T3-L1 cells overexpressing nuc-FoxO1 up-regulate brown-related mitochondrial markers such as UCP1, Drp1 and SOD2 proteins (Fig 6F and Fig 6G). In line with the enhancement of the mitochondrial functionality coupled with the UCP1 induction, we detected enhanced oxygen consumption (Fig 6H) and increased uncoupling ratio (Fig 6I) exclusively in adipocytes overexpressing nuc-FoxO1. Reduced ΔΨM in nuc-FoxO1 cells further corroborates the occurrence of a brown fat-like function in white adipocytes (Fig 6J).

## DISCUSSION

Defective mitochondrial functionality and visceral adipose tissue expansion are crucial footprints of age-related metabolic disorders [26-27]. Dietary or nutrient restriction such as fasting represents a strong physiological stimulus that links *geroprotection* to improved mitochondrial metabolism and fat mass remodeling [6-7]. The function of mitochondria as sensors of metabolic imbalances and mediators of specific cellular stress responses that can impact longevity has recently collected increased attention [28].

Herein we have demonstrated that upon nutrient deprivation (i.e. fasting), concomitantly to the previously observed induction of lipid oxidative genes [4, 17], white/beige adipose cells undergo mitochondrial reorganization and build-up a brown-like phenotype. In particular, nutrient limitation increases ^mt^DNA/^n^DNA-encoded OxPHOS complexes ratio, which represents an early response that ultimately leads to OxPHOS machinery reorganization and enhanced mitochondrial functionality. Similarly, Mouchiroud and coworkers revealed that mitonuclear protein imbalance occurs during nutrient-restriction mimicking conditions (i.e. by increasing NAD^+^ levels) and they have identified this response as a conserved longevity pathway [16].

In our work we reported increased mitochondrial fragmentation in starved white/beige fat cells. Such mitochondrial fragmentation may be an adaptive strategy to more efficiently attain neo-synthesized mitochondrial proteins such as UCP1. In accordance with this assumption, Wikstrom and co-workers, discussed that mitochondrial fragmentation is a helpful mitochondrial response to store neo-synthesized UCP1 in hormonally stimulated brown adipocytes [22]. Interestingly, by exposing starved adipocytes to nutrient overload we detected a reduced P:O ratio. This result strongly suggests that UCP1 induction, associated with improved OxPHOS machinery, was effective in its uncoupling function. Our data propose that a temporarily restricted fasting (1 or 2 days) is effective in *tuning-up* white adipose cells in a high oxidative mode making them more efficient in buffering postprandial nutrient excess. In line with this idea, 6 months randomized clinical trial showed that fasting for two non-consecutive days per week results in reduced body weight and fat mass as well as improved metabolic profile [6-7].

Emerging is the induction of UCP1 as adaptive response to suppress ROS production in adipose cells. Remarkably, exacerbation of mitochondrial damage by the inhibition of UCP1 supports this idea [29-33]. In line with our previous work [34], these data suggestively put the *browning program* in a stress defensive response of white fat cells, whereas the higher level of antioxidant proteins harbored by BAT could partially clarify the absence of mitochondrial responses to nutrient deprivation. Mitochondrial UCP1 and SOD2 induction could restrain excessive ROS production deriving from the boosted oxidative metabolism of white fat cells exposed to nutrient shortage. Furthermore, the chemical or genetic strategy enhancing antioxidant defenses (NAC supplementation and SOD2 overexpression) limits the mitochondrial rearrangement of white/beige adipocytes after NR. It is plausible that dietary antioxidants supplementation may affect ROS-dependent physiological processes that are important for metabolic homeostasis. Accordingly, many human clinical studies failed to show benefits of dietary antioxidants in treating age- or obesity-associated diseases [35-38]. Several works have shown that specific stress responses initiated by mitochondria that experience functional impairments could extend lifespan, and ^mt^ROS are key mediators of these responses [23, 28]. In a previous work we showed that ^mt^ROS are up-stream inducers of mitochondrial oxidative genes in white adipose cells during NR preventing cell death [4]. Here, we have given further proof of the importance of ^mt^ROS in triggering adaptive responses in adipocytes as they activate a unique pattern of stress defensive genes leading to the acquirement of a brown-like phenotype. As reviewed by Blagosklonny, a weak stress such as calorie restriction prolongs lifespan and delays the occurrence of age-related metabolic disorders. Such hormetic response occurs when the target of rapamycin (TOR) is deactivated [39]. Accordingly, in our previous work we demonstrated that in adipocytes the metabolic adaptation to nutrient restriction is associated with the silencing of the TOR pathway [17]. Based on this evidence, we can place the herein deciphered ^mt^ROS-mediated signaling in a mitochondrial hormesis (mitohormesis) program that ultimately improves adipocyte metabolism.

Notably, we demonstrated that white/beige adipocytes directly sense nutrient scarcity and WAT mass is consequently reduced in a way that is independent of hormone-mediated cAMP/PKA signaling. Our findings also reveal that the different redox state between white and brown fat could elucidate the diverse response to fasting and emphasize that this dietary approach is healthful for WAT shifting mitochondrial activity toward a brown-like metabolism. The metabolic adaptability of white/beige adipocytes to nutrient deprivation could be allowed through activation of ^mt^ROS/^n^FoxO1 axis, a peculiar signaling that is commonly placed in longevity pathways [15].

Overall our data could provide a molecular mechanism that links the beneficial effects of dietary restriction (i.e. fasting or intermittent fasting) to WAT reprogramming and lifespan.

## METHODS

### Animals and treatments

We conducted all mouse experimentations in accordance with accepted standard of humane animal care and with the approval by relevant national (Ministry of Health) and local (Institutional Animal Care and Use Committee, Tor Vergata University) committees. CD1 and C57BL/6 adult male mice were purchased from Harlan Laboratories S.r.l. (Urbino, Italy).

For *in vivo* experiment, twelve CD1 mice were randomly divided into two age-matched (4 months old) groups: *ad libitum* fed (Ctr) by standard diet and 20 h fasted (NR). Two C57BL/6 male mice per group were sacrificed to compare mitochondrial OxPHOS proteins in mitochondrial-derived eWAT and BAT at different ages (1, 3 and 6 months old). Adult C57BL/6 mice were randomly divided into three age-matched (4 months old) groups: *ad libitum* fed (Ctr) by standard diet; 20 h fasted and SR59230A (Santa Cruz Biotechnology, Heidelberg, Germany) treated mice. In this period each NR mouse had free access to water and was kept on a 12:12 h light:dark cycle. For treatment with the selective β_3_-adrenoreceptor antagonist, mice were intraperitoneally (ip) injected with 10 mg/kg of SR59230A dissolved in a physiological saline solution. Both *ad libitum* and NR group was ip injected with saline solution. SR59230A was injected 6 h prior and at the beginning of fasting experiment. Mice were sacrificed by cervical dislocation, and tissue samples were adequately collected. For total protein and mRNA extraction, epididymal (eWAT), inguinal (iWAT) and brown (BAT) adipose tissue were explanted and immediately processed.

### Cell lines, differentiation, primary cell isolation, transfections and treatments

3T3-L1 cell lines were purchased from ATCC (Manassas, VA, USA). T37i cell line was gently provided by Prof. Marc Lombes (Inserm U693, Paris, France) and X9 cells were gently provided by Prof. Bruce Spiegelman (Harvard Medical School, Boston, MA, USA). NR experiments were carried out as previously described [4, 17]. 3T3-L1-white adipocytes were cultured as previously described [4]. T37i cells were cultured and differentiated as described by Nakae et al., 2008 [40]. Immortalized X9-beige cell line was cultured and differentiated as reported by Ye et al. [9]. All experiments were performed in fully differentiated adipocytes (day 8).

For isolation of primary adipocytes, the stromal-vascular fraction of the epididymal (eWAT), inguinal (iWAT) and brown (BAT) fat pads of 5 weeks old male C57BL/6 mice were prepared and differentiated for 8 days, as reported by Fisher at al., 2012 [41].

Fully differentiated 3T3-L1-white or X9-beige adipocytes were transfected with FoxO1 (Santa Cruz Biotechnology) or scramble siRNAs (Santa Cruz Biotechnology) by using DeliverX Plus kit (Affymetrix, Santa Clara, CA, USA). Alternatively, they were transfected with SOD2 cDNA pcDNA3.1 plasmid or pCMV-DsRed plasmid (Clontech Lab, Mountain View, CA, USA) by using Turbofect Transfection Reagent (Thermo Scientific, Rockford, IL, USA). Adipocytes were subjected to NR 48 h after transfection. Plasmid nuc-FoxO1 pCMV5 (Addgene #12143) and cyt-FoxO1 (7KQ mutant, gently provided by Prof. Accilli D., Dept. of Medicine, Columbia University, New York, NY) were used to transfect 3T3-L1 cells.

Nutrient refill (re-feed) was performed by culturing starved adipocytes in complete culture medium (CM) for 1 h. Isoproterenol (Sigma-Aldrich) and N-acetyl cysteine (NAC) (Sigma-Aldrich) were dissolved in PBS and added in culture medium at final concentration of 10 μM and 2 mM, respectively. NAC was added 1 h prior to NR and maintained throughout the experiment. Rotenone (Sigma-Aldrich) was solubilized in DMSO and added in culture medium at final concentration of 0.1 μM.

### Western blotting and OxPHOS protein ratio

Western blotting analysis was performed as previously described [17]. OxPHOS protein ratio was evaluated by Western blot calculating the stoichiometric ratio between nuclear-encoded and mitochondrial-encoded proteins after densitometric analysis of the immunoreactive bands. Protein concentration was determined by the method of Lowry.

### RT-qPCR and OxPHOS gene expression analysis

RT-qPCR analysis was carried out as previously described [4]. To calculate OxPHOS gene expression ratio, nuclear-encoded OxPHOS mRNA levels were compared to mitochondrial-encoded OxPHOS mRNA. The relative mRNA levels were determined by using the 2-^ΔΔCt^ method and were normalized to β-actin. ΔCt values were used to generate Heat Map by Excel software.

### Nuclear, cytoplasmic and mitochondrial fractioning

Nuclear and cytosolic fractions were obtained from cells and mice tissues using a commercially available NE-PER® extraction kit (Thermo Scientific). Mitochondria from differentiated adipocytes and eWAT were obtained as described by Wieckowski et al. [42].

### Confocal microscopy

Olympus Fluoview 1000 confocal laser scanning system was used for colocalization experiments. The 488 nm laser was used to excite MTG and AlexaFluor 488-conjugated secondary antibody (Life Technologies). The 543 nm laser was used for detection of MTR and AlexaFluor 568 conjugated secondary antibody (Life Technologies). A 63X objective was used for all images. Nuclei were stained with Hoechst 33342 (10 μg/ml). Mitochondrial morphology analysis was performed by using Mitophagy Macro Plugin (ImageJ Software), and mitochondria area, perimeter and circularity were reported.

### Cell respiration and ATP production

Oxygen consumption rate was determined in intact 3T3-L1-white and X9-beige adipocytes. Briefly, at the end of differentiation, cells were either starved for 4 h or cultured in complete culture medium. Real-time oxygen consumption measurement was determined at 30 °C, for 6 min by Oxygraph Plus oxygen electrode system (Hansatech Instruments Ltd, Norfolk, UK). Nutrient refill was performed by resuspending starved adipocytes in complete culture medium. Oxygen consumption was normalized for cell number. After cell respiration analysis, ATP level was detected by using ATP Bioluminescence assay kit (Roche Diagnostics) and values were normalized for cell number. To evaluate cellular uncoupling, we conventionally normalized oxygen consumption for ATP content.

### Cytofluorimetric analysis of mitochondrial membrane potential, mass and ROS

Mitochondrial ROS, transmembrane potential and number were detected by incubating cells with the fluorescent probe MitoSOX RED (5 μM), MitoTracker Red CMX ROS (250 nM), Tetramethylrhodamine Ethyl Ester Perchlorate (TMRE, 20 nM), MitoTracker Green (250 nM) or Nonyl Acridine Orange (NAO, 10 nM) (Life Technologies) for 30 min at 37 °C. Subsequently, cells were collected and used for cytofluorimetric analyses by FACScalibur instrument (Beckton and Dickinson, San José, CA, USA). Changes in mitochondrial membrane potential were expressed as Red-to-Green ratio [43].

### Statistical analysis

The results are presented as means ± S.D. Statistical evaluation was conducted by ANOVA, followed by the post Student-Newman-Keuls, by using GraphPad Prism 5 Software (GraphPad Software).

## SUPPLEMENTAL FIGURES


