# Erratum: Mitochondrial Hormesis links nutrient restriction to improved metabolism in fat cell

**DOI:** 10.18632/aging.101003

**Published:** 2016-07-19

**Authors:** Daniele Lettieri Barbato, Giuseppe Tatulli, Katia Aquilano, Maria R. Ciriolo

Aging (Albany NY) 2015; 7(10): 869-881.

PMCID: PMC 4637211 PMID: 26540513

The last name of first author of this article is provided wrong on PubMed: **Barbato DL**

http://www.ncbi.nlm.nih.gov/pubmed/?term=Mitochondrial+Hormesis+links+nutrient+restriction+to+improved+metabolism+in+fat+cell.

The correct citation of this author for PubMed is: **Lettieri Barbato D.**

