# Supplementary material for: Mitochondrial Hormesis links nutrient restriction to improved metabolism in fat cell
Source: Aging (Albany NY). 2015 Oct 28;7(10):869–81. doi: 10.18632/aging.100832 (PMC4637211; doi:10.18632/aging.100832)
Supplement: Supplementary file 1 [file aging-07-869-s001.pdf]

# Mitochondrial hormesis links nutrient restriction to improved metabolism in fat cells

**Supplementary Material**

**LIST OF SUPPLEMENTAL INFORMATION**

Supplemental Figures:

Figure S1. Mitonuclear OxPHOS proteins and genes expression.

Figure S2. Brown fat-like change and adipose tissue remodelling upon NR.

Figure S3. Mitochondrial antioxidant proteins and redox state of fat cells.

Figure S4. NR-like responses upon mitochondrial complex I inhibition

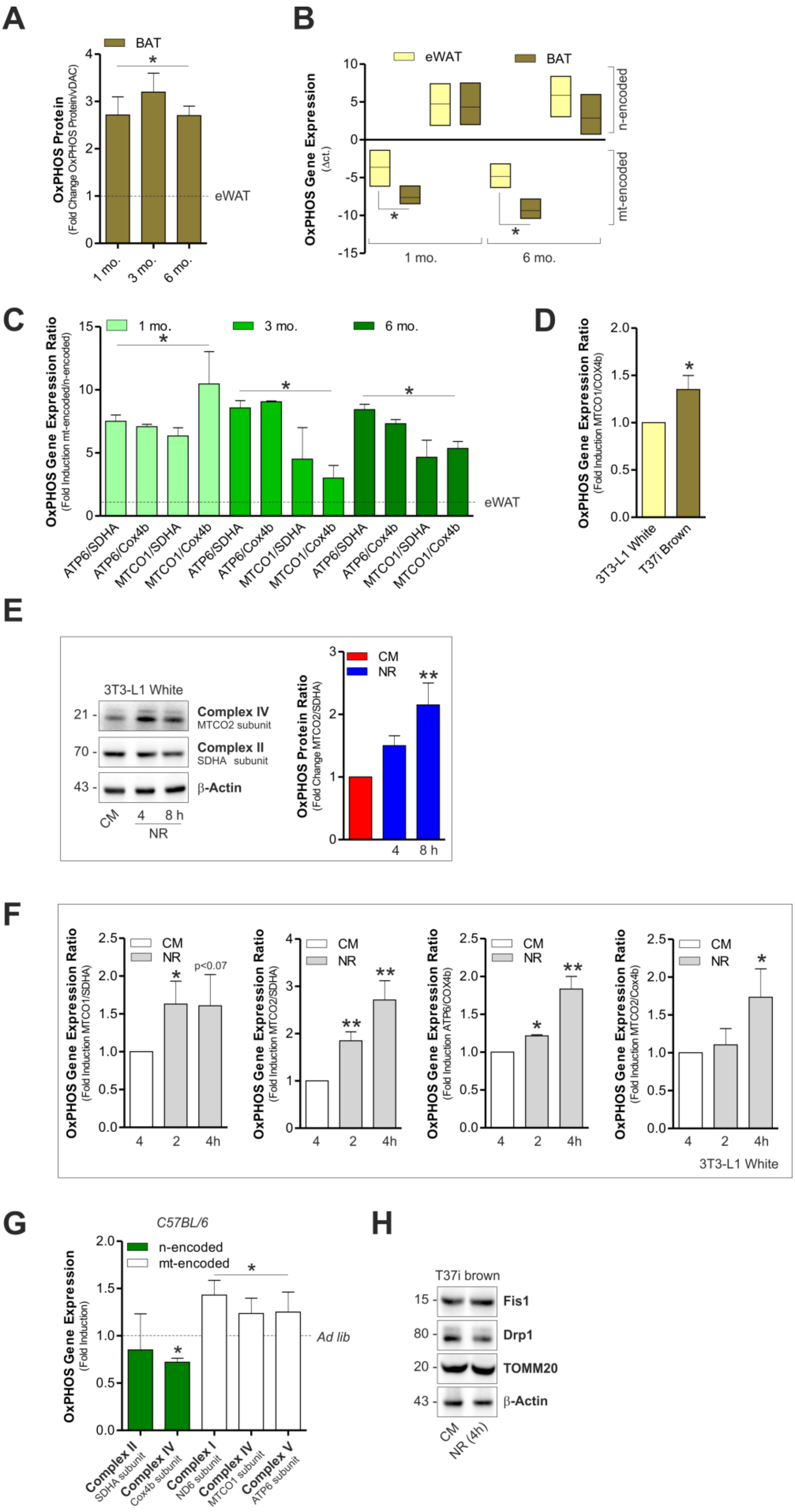
A
BAT
OxPHOS Protein
(Fold Change OxPHOS Protein/vDAC)
eWAT
1 mo.
3 mo.
6 mo.
B
eWAT
BAT
OxPHOS Gene Expression
(Δct.)
n-encoded
mt-encoded
1 mo.
6 mo.
C
1 mo.
3 mo.
6 mo.
OxPHOS Gene Expression Ratio
(Fold Induction mt-encoded/n-encoded)
eWAT
ATP6/SDHA
ATP6/Cox4b
MTCO1/SDHA
MTCO1/Cox4b
D
OxPHOS Gene Expression Ratio
(Fold Induction MTCO1/COX4b)
3T3-L1 White
T37i Brown
E
3T3-L1 White
Complex IV
MTCO2 subunit
Complex II
SDHA subunit
β-Actin
CM
NR
4
8 h
OxPHOS Protein Ratio
(Fold Change MTCO2/SDHA)
F
CM
NR
p<0.07
OxPHOS Gene Expression Ratio
(Fold Induction MTCO1/SDHA)
(Fold Induction MTCO2/SDHA)
(Fold Induction ATP6/COX4b)
(Fold Induction MTCO2/Cox4b)
4
2
4h
3T3-L1 White
G
C57BL/6
n-encoded
mt-encoded
OxPHOS Gene Expression
(Fold Induction)
Ad lib
Complex II
SDHA subunit
Complex IV
Cox4b subunit
Complex I
ND6 subunit
Complex IV
MTCO1 subunit
Complex V
ATP6 subunit
H
T37i brown
Fis1
Drp1
TOMM20
β-Actin
CM
NR (4h)

**Figure S1. Mitonuclear OxPHOS proteins and genes expression**

(**A**) Densitometric analysis of OxPHOS protein levels in adipose depots (see Figure 1A) (n=2 mice per group).

(**B**) OxPHOS gene expression, reported as Δct, analysed by RT-qPCR (see Figure 1C) (n=2 mice per group).

(**C**) OxPHOS gene expression, reported as fold induction *vs* eWAT, analysed by RT-qPCR (see Figure 1C) (n=2 mice per group).

(**D**) OxPHOS gene expression ratio evaluated by calculating the ratio between $^{mt}$DNA-encoded and $^{n}$DNA-encoded OxPHOS genes after RT-qPCR.

(**E**) OxPHOS protein ratio (*right panel*) evaluated by calculating the ratio between $^{mt}$DNA-encoded (MTCO2) and $^{n}$DNA-encoded (SDHA) proteins after Western blot (see Figure 1D).

(**F**) OxPHOS gene expression ratio evaluated in 3T3-L1 cells by calculating the ratio between $^{mt}$DNA-encoded (MTCO1, ATP6 and MTCO2) and $^{n}$DNA-encoded (SDHA and Cox4b) OxPHOS genes after RT-qPCR (see Figure 1E).

(**G**) $^{mt}$DNA-encoded and $^{n}$DNA-encoded OxPHOS mRNAs measured by RT-qPCR (n = 4 mice per group).

(**H**) Fis1, Drp1 and TOMM20 proteins evaluated by Western blot analysis.

Actin staining served as loading control. Bar graphs are expressed as mean ±S.D. (n=4; *p<0.05; **p<0.01 *vs* CM).

NR: nutrient restriction; CM: complete medium.

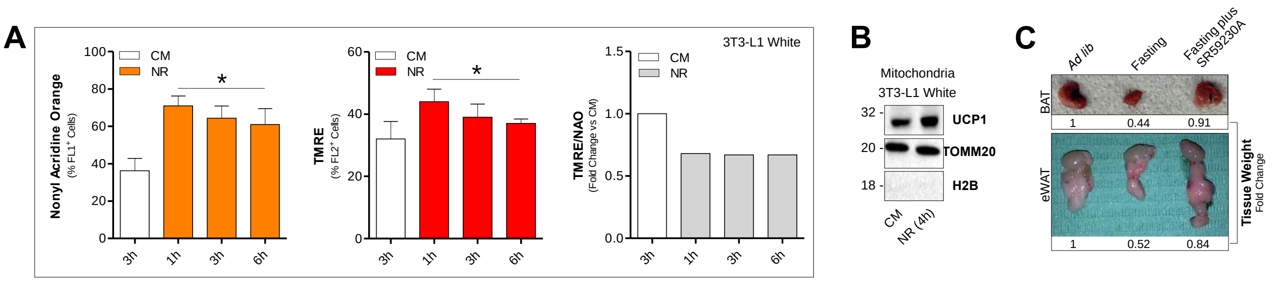


**Figure S2. Brown fat-like change and adipose tissue remodelling upon NR**

(**A**) Cytofluorimetric detection of mitochondrial amount and membrane potential after Nonyl Acridine Orange (NAO) and Tetramethyl Rhodamine Ester (TMRE) staining, respectively. Polarized mitochondria determined by calculating TMRE-to-NAO ratio (See Figure 2F, G and H).

(**B**) UCP1 protein level evaluated in mitochondrial extracts by Western blot analysis (see Figure 3B).

(**C**) eWAT and BAT explanted from *ad libitum* fed or 20 h fasted C57BL/6 mice treated or not with the selective $\beta_3$-receptor antagonist SR59230A (n=4 mice per group).

TOMM20 and H2B staining served as loading controls or for assessing the purity of protein fraction.

Bar graphs are expressed as mean ±S.D. (n=3; *p<0.05 *vs* CM).

NR: nutrient restriction; CM: complete medium.

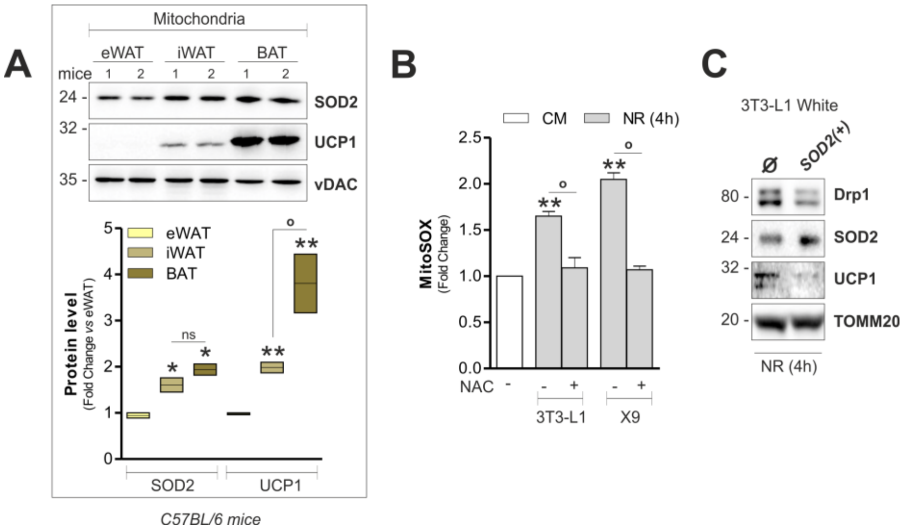


**Figure S3. Mitochondrial antioxidant proteins and redox state of fat cells.**

(**A**) Western blot (*upper panel*) and relative densitometric analysis (*bottom panel*) of SOD2 and UCP1 in crude mitochondria (n = 4 mice per group).

(**B**) Cytofluorimetric detection of mitochondrial ROS after staining with MitoSOX in cells treated or not with NAC.

(**C**) Western blot analysis of Drp1, SOD2 and UCP1 in cells transfected with a SOD2 cDNA [SOD2(+)] or with an empty vector (Ø).

TOMM20 and vDAC staining served as loading controls. Bar graphs are expressed as mean ±S.D. (n=4; *p<0.05; **p<0.01 vs CM; °p<0.05).

NR: nutrient restriction; CM: complete medium.

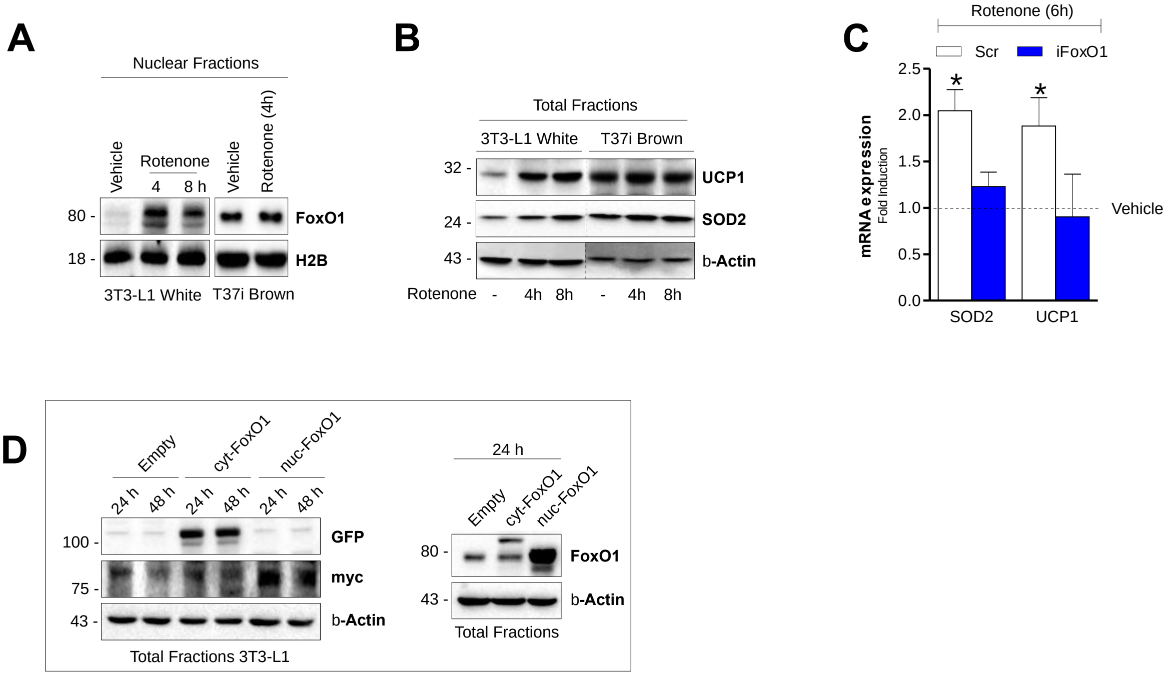


**Figure S4. NR-like responses upon mitochondrial complex I inhibition**

(**A**) Western blot analysis of FoxO1 in nuclear fractions of white and brown adipocytes treated with rotenone.

(**B**) Western blot analysis of UCP1 and SOD2 in total fractions of white and brown adipocytes treated with rotenone.

(**C**) SOD2 and UCP1 mRNA expression analysed by RT-qPCR in 3T3-L1 white adipocytes treated with rotenone.

(**D**) Western blot analysis of GFP-tagged, myc-tagged FoxO1 (*left panel*) and untagged FoxO1 (*right panel*) in 3T3-L1 cells transfected with nuc-FoxO1, cyt-FoxO1 cDNAs or empty vector.

Actin and H2B served as loading controls. Bar graphs are expressed as mean ±S.D. (n=4; *p<0.05 *vs* vehicle).
